# Thirteenth Annual Meeting of the Mississippi Valley Association

**Published:** 1857-03

**Authors:** 


### Proceedings of Societies.

---

### THIRTEENTH ANNUAL MEETING OF THE MISSISSIPPI VALLEY ASSOCIATION.

The association met according to adjournment in the Dental College, at 10 o'clock, Wednesday, February 25, 1857.

Present: Drs. C. Bonsall, J. Taylor, J. Richardson, E. Osmond, G. Watt, of Cincinnati, O.; Dr. A. G. Stipher, Decatur, Ill.; Dr. J. W. Baxter, Warsaw, Ky.; Dr. G. W. Keely, Oxford, O.; Dr. J. P. Ullery, Rising Sun, Ind.; Dr. H. R.

Smith, Terre Haute, Ind.; Dr. J. Taft, Xenia, O.; Dr. H. McCollum, Augusta, Ky.; Dr. J. G. Hamill, Xenia, O.

The President and Vice-President being absent, on motion of Dr. Bonsall, Dr. H. McCollum was unanimously elected President pro tem.

The minutes of the last meeting were read, and the report of the Executive Committee called for, which was presented and adopted as follows:—

Report of Executive Committee.—Your Committee would respectfully submit to the association the following report on the order of business:

I. Report of officers.

II. Report of committees.

III. Addresses and Essays.

IV. Miscellaneous business:

1. Is "annealed foil," as now used, preferable to blocks for filling; and does it weld as perfectly as crystal gold?

2. Is the exalted sensibility of decayed teeth the result of inflammation?

3. What constitutional conditions will most predispose the teeth to decay?

4. What has been the course pursued, and what the success in the treatment of alveolar abscess?

5. How does crystal gold stand the test of time?

6. What success has been obtained in fang filling?

7. What is the best manner for filling fangs?

8. Is it desirable, as a general rule, to preserve the fangs of teeth for the reception of artificial substitutes?

9. What conditions of the mouth should determine us in the choice of materials for taking impressions for full upper dentures?

10. What are the indications for the removal of the deciduous teeth?

V. The election of officers.

J. Taft,

J. P. Ullery,

J. Richardson. } *Committee.*

The Treasurer presented his report and requested that a committee be appointed to audit the same.

TREASURER'S REPORT.—The Treasurer would respectfully report the following as the state of his accounts, and request that an auditing committee be appointed to examine them:

| | | |
|---|---|---|
| Balance in the treasury at last report . . . . | | $11 02 |
| Received since, for initiation fees . . . . . . . | | 50 00 |
| " " Annual dues . . . . . . | | 45 00 |
| | | $106, 02 |
| Credit by cash paid janitor . . . . . . . . | 5 00 | |
| " " Dr. Goddard, for woodcuts . | 16 00 | |
| " " E. Babb, for reporting . . | 7 00 | |
| " " Postage, Stationery, etc. . . | 1 02 | |
| | 29 02 | |
| Leaving a balance in the treasury of . . . . | | $77 00 |

He would also refer to having notified, as directed, delinquent members, several of whom have responded by forwarding their dues to the association.

CHARLES BONSALL, *Treas.*

In accordance with the above request, the President appointed Drs. Taft, Taylor and Ullery an auditing committee, who presented the following report:

Your committee respectfully report that they have examined the Treasurer's account, and find the same to be correct.

J. TAFT,
J. TAYLOR, } *Committee.*
J. P. ULLERY.

The Corresponding Secretary presented the following report which was accepted:

REPORT OF CORRESPONDING SECRETARY.—The Corresponding Secretary, would report that one or two gentlemen have written in relation to membership. In reply, the Secretary informed them of the terms of membership, etc. The individuals are not present, nor has the initiation fee been forwarded so that their names can be acted on. I have also a letter from the President and one from Dr. A. M. Leslie, both resigning their membership on account of having retired from the profession. These letters are herewith submitted to the association. JAS. TAYLOR, *Cor. Sec'y.*

On motion of Dr. Bonsall, resolved that all members of the dental and medical professions who are or may be present, be invited to participate in the discussions of the society.

The committee on microscopic examinations had no report. On motion of Dr. Watt, said committee was discontinued.

The committee on dental progress had no report.

On motion, adjourned till 3 o'clock, P. M.

AFTERNOON SESSION.—The association met according to adjournment. The minutes were read and adopted.

The committee on publication of Essay presented the following report, and requested that a committee be appointed to audit the same.

REPORT OF COMMITTEE ON PRIZE ESSAY.

Your committee respectfully report that they had the Prize Essay stereotyped, and have issued three thousand copies to fill the orders received at the last meeting of the association. These have been distributed to the subscribers. The cost of stereotyping, as per bill, is $57. 82, to meet which the committee borrowed the amount from the Treasurer of the association. The cost of printing the three thousand copies was $126, 50; making the total expense $184, 32. To meet this there has been collected on subscription $162, 25, leaving a deficit of $22, 07. To meet this there is yet to collect on subscriptions, $30, 75, making a surplus of $7, 68.

J. TAFT,<br>J. P. ULLERY, } *Committee.*<br>JAS. TAYLOR.

Drs. Bonsall, Stipher and Baxter were appointed a committee to audit the above account, who, after examination reported as follows:

The auditing committee, having examined the foregoing accounts find them correct.

C. Bonsall,

A. G. Stipher, } *Committee.*

J. W. Baxter.

On motion of Dr. Bonsall, the resignations of Drs. Leslie and Newbrough, reported by the Corresponding Secretary, were accepted.

A resolution was offered by Dr. Richardson, and adopted as follows:

*Resolved*, That after the close of the discussion of questions regularly before the association, it shall be the privilege of any member to relate any case or cases of interest that may have come under his observation; also, any mode or modes of practice not generally known to the profession, and not contemplated by the subjects previously discussed.

Essays and addresses being called for, none were read.

The essayists were excused and their appointments continued.

On motion of Dr. Bonsall it was resolved that the acting secretary at each meeting be exempted from his annual dues to the society.

The association proceeded to the miscellaneous business; and the discussion of No. 1 occupied the remainder of the session.

SECOND DAY—FEB. 26, 1857.

Morning Session.—The association met according to adjournment. The Minutes were read and adopted. The discussion of No. 1 was resumed, and continued during most of the morning session.

On motion of Dr. Watt, Mr. James Leslie, foil maker, of Cincinnati, was requested to participate in the discussions, who accepted the invitation, and, after a number of interesting remarks, demonstrated the proper method of, and the changes produced by annealing foil. Mr. L. prepares a wire frame of rectangular shape, of a diameter a little larger than the leaf of foil, the ends of the wire projecting far enough to form a convenient handle; this rectangle is bisected diagonally by two small wires, to prevent the foil from falling through it. On this frame a leaf of foil is laid, and an alco hol flame is carried under it by the hand, till it is heated to low redness.

On motion of Dr. Taft, the thanks of the association were returned to Mr. Leslie for his interesting remarks and demonstrations.

The Committee on Membership presented the following names as candidates for membership: Dr. H. A. Smith, Oxford, O.; Dr. C. W. Spalding, St. Louis, Mo.; Dr. A. G. Philips, Barnesville, O.; Dr. Chas. Silsbee, Cincinnati, O.; Dr. J. R. Walker, Richmond, Texas; all of whom were unanimously elected. Drs. Taylor and Keely were appointed a committee to notify the gentlemen of their election.

On motion of Dr. Taft, the exhibition of instruments, specimens, etc., was made the order of business for 3 o'clock, P. M.

The forenoon session being nearly spent, the discussions on miscellaneous business were postponed for the time, and the association proceeded to the election of officers, which resulted as follows:

*President.*—Dr. C. W. SPALDING.

*Vice-President.*—Dr. H. M'COLLUM.

*Corresponding Secretary.*—Dr. J. TAFT.

*Recording Secretary.*—Dr. G. WATT.

*Treasurer.*—Dr. C. BONSALL.

*Executive Committee.*—Dr. J. RICHARDSON, Dr. J. TAFT, Dr. J. G. HAMILL.

*Committee on Membership.*—Dr. J. TAYLOR, Dr. G. W. KEELY, Dr. J. P. ULLERY.

*Committee on Dental Progress.*—Dr. J. RICHARDSON, Dr J. TAFT, Dr. H. R. SMITH.

On motion, adjourned to meet at 3 o'clock, P. M.

AFTERNOON SESSION.—Met according to adjournment.

The Minutes were read and adopted. President M'Collum appointed Dr. Taft to conduct the President-elect to the chair, on taking which, President Spalding thanked the society for the honor conferred, and made some highly interesting remarks on the importance of dental associations, which were warmly appläuded.

The order of business being the exhibition of instruments, morbid specimens, etc., Mr. J. W. Sharp exhibited "Lawson's patent Furnace and Blow-pipe;" Dr. Bonsall exhibited plaster models, illustrating the case of morbid growth of the gums, reported in the October number of the Dental Register; Dr. Taft exhibited "Mallett's plate punch," some highly finished specimens of teeth by Messrs. Jones, White and M'Curdy, a warm air syringe for drying cavities, some morbid specimens, presented by Dr. E. Collins, of Connersville, Ind., including a case of exostosis, complicated with osseous union of the roots of two molars, a case of osseous union of the crowns of two deciduous teeth, and a tooth much colored, apparently by an amalgam filling, and also a specimen copy of "Rich's Dental Record;" Dr. I. B. Branch exhibited his instruments for producing local anasthæsia; Dr. J. M. Brown exhibited some specimens of very beautiful French teeth; Mr. J. T. Toland exhibited a copy of Dr. Waters' "Complete Dental Register," and Mr. S. Palmer exhibited quite a variety of beautiful teeth, manufactured by Messrs. Orum and Armstrong.

On motion of Dr. Taylor, the thanks of the association were returned to each and to all who were instrumental in favoring the society with the interesting exhibition just witnessed.

The association proceeded to the discussion of No. 2 of Miscellaneous Business. At the close of the consideration of this subject, on motion of Dr. Taft, Nos. 6 and 7 were made the order of the day for 7 o'clock this evening.

On motion of Dr. Bonsall, adjourned to meet at 7 o'clock this evening.

Evening Session.—Met according to adjournment. The Minutes were read and adopted. The order of the day occupied the entire session.

On motion of Dr. Bonsall, adjourned till 9 o'clock to-morrow morning.

### THIRD DAY.

Met according to adjournment. The discussion of Miscellaneous Business was resumed; Nos. 3 and 4 occupying the most of the session.

On motion of Dr. Taft, it was resolved the discussions close at 12 o'clock.

Dr. Taylor offered the following resolutions, which were adopted:

*Resolved*, That ten dollars be appropriated toward reporting our proceedings.

*Resolved*, That five dollars be appropriated to pay the janitor for his services during our sessions.

On motion ot Dr. Taft,

*Resolved*, That the stereotype plates of the Prize Essay be placed in charge of the Secretary, and that he be authorized to issue any required edition of the essay, and dispose of them at the rates fixed by the society at its last meeting.

On motion of Dr. Bonsall, the thanks of the association were returned to Dr. M'Collum, for the faithful and satisfactory discharge of his duties as President.

On motion, adjourned to meet at this place, on Wednesday, Feb. 17th, 1858, at 10 o'clock, A. M.

---

